# A novel epileptic seizure prediction model based on Cox-Stuart and Optuna

**DOI:** 10.3389/fneur.2025.1624873

**Published:** 2025-10-16

**Authors:** Xizhen Zhang, Xiaoli Zhang, Fuming Chen

**Affiliations:** ^1^Medical Security Center, The 940th Hospital of the Joint Logistics Support Force of the Chinese People's Liberation Army, Lanzhou, China; ^2^Gansu University of Traditional Chinese Medicine, Lanzhou, China

**Keywords:** epilepsy prediction, Cox-Stuart, Optuna, CNN, CNN-BiLSTM

## Abstract

**Objectives:**

In order to more accurately predict whether patients with intractable epilepsy are about to develop seizures, this paper proposes an epilepsy prediction model.

**Methods:**

When the amount of targeted patient data is small, A Cox-Stuart and Convolutional Neural Network and Bi-directional Long Short-Term Memory (Cox-Stuart-CNN-BiLSTM) model based on multi-patient epilepsy prediction is proposed, which aims to capture common features of epileptic seizures by integrating EEG signal data from multiple patients to train the model. When there is enough data for targeted patient, an Optuna and Convolutional Neural Network and Bi-directional Long Short-Term Memory (Optuna-CNN-BiLSTM) model based on independent patient epilepsy prediction is proposed, which can train the model for EEG data of individual patients, aiming to better match physiological characteristics and seizure patterns of targeted patient.

**Results:**

The accuracy of the test set for multi-patient is 0.9992, the sensitivity is 0.9996, and the specificity is 0.9988; the average accuracy of the test set for independent patient is 0.9996, the sensitivity is 0.9995, and the specificity is 1.0000.

**Conclusions:**

It can be proved that the method proposed in this paper has good experimental results.

## 1 Introduction

Epilepsy is a chronic brain disorder, and its seizures are caused by sudden abnormal discharges of neurons in the brain, with complex causes. The International League Against Epilepsy guidelines summarize the following causes: genetic structural causes, infectious causes, structural causes, immune causes, metabolic causes, and unknown causes (1). Currently, there are approximately 50 million people with epilepsy worldwide, and it affects individuals of all ages. Although various treatment methods for epilepsy have been proposed, about 30% of patients still experience recurrence (2) The occurrence of epilepsy is sudden and recurrent, causing significant physical and emotional distress to both patients and their families. Although epileptic seizures can be predicted based on an epilepsy diary, the accuracy is < 50% (3) Therefore, effective methods for predicting epilepsy are of great significance. Electroencephalography (EEG) is one of the most important methods for studying epilepsy and capturing changes in brain electrical activity. It is used to examine the brain electrical activity changes that cause epilepsy and to identify potential epileptic seizures (4, 5) Currently, publicly available epilepsy datasets are collected using EEG signals.

Pan et al. (6) utilized raw EEG data, as well as EEG data processed by Fast Fourier Transform (FFT), Short-Time Fourier Transform (STFT) and Discrete Wavelet Transform (DWT) as inputs for a Convolutional Neural Network (CNN). They employed a feature fusion mechanism to integrate the learned features, achieving an accuracy of over 99%. However, their experiment did not include a test dataset, and the results were derived from cross-validation, making it impossible to determine generalization ability of the model. Takahashi et al. (7) applied high-pass, low-pass, and notch filtering to the raw data. They then used an Autoencoder (AE) to define data with a high AE error during interictal periods (inter-ictal) of more than 10 s as non-epileptic but abnormal data. This data was then used as input for a CNN, reducing the false alarm rate to 0.034/h, which is one-fifth of the false alarm rate of the original CNN. Preprocessing steps are not always necessary, for example, Golmohammadi (8) directly used Linear Frequency Cepstral Coefficients (LFCCs) and their first and second derivatives for feature extraction, then input them into a Long Short-Term Memory network (LSTM). Although specificity was higher than 90%, sensitivity was < 35%. Jana et al. (9) used the Non-dominated Sorting Genetic Algorithm II (NSGA-II) to select data from the three optimal channels, reducing computational complexity. They directly used a One-Dimensional Convolutional Neural Network (1D-CNN) for feature extraction and classification, achieving accuracy, sensitivity, and specificity of over 96%. However, their experiment did not include a test dataset, and due to high computational time complexity, only data from five patients were selected for channel selection and classification. Li et al. (10) demonstrated that a CNN with a Waxman similarity graph achieved the highest accuracy. Over 98% of the EEG 1-second epochs were correctly classified into ictal periods(ictal), pre-ictal periods(pre-ictal), or inter-ictal. However, their dataset was relatively small. Toraman et al. (5) compared three pre-trained CNN models: VGG16, ResNet, and DenseNet. They used spectrogram images to distinguish between pre-ictal and inter-ictal states and found that the ResNet model performed the best, with an accuracy of 90.32%, sensitivity of 91.05%, and specificity of 89.76%. This method addresses the issue of limited experimental data. Considering the sequential feature of EEG signals, Aslam et al. (11) used a Convolutional Neural Network and Long Short-Term Memory network (CNN-LSTM) hybrid model to classify EEG signals and predict seizures, achieving an accuracy of 94%, sensitivity of 93.8%, and specificity of 91.2%. To enhance the handling of long-term temporal dependencies, Ma et al. (12) introduced a Cross-Channel Feature Fusion-based CNN-BiLSTM model. This model integrates attention mechanisms and channel fusion to effectively manage long-term temporal signals while reducing computational complexity. On the CHB-MIT dataset, it achieved an accuracy of 94.83% and sensitivity of 94.94%, but on the Bonn dataset, the accuracy and sensitivity were below 80%. Indurani et al. (13) used a Time-Attention CNN with LSTM, achieving accuracy and sensitivity of over 94% on both the CHB-MIT and Bonn datasets. Most existing literature does not mention the choice of data partitioning methods for epilepsy datasets, although different partitioning methods can yield different experimental results. In addition to the above literature, it is worthwhile to learn from the ideas of other research directions. He et al. (14) proposed a Fitness count-based red deer algorithm that can determine the optimal weights of the features as well as the optimal parameters of the model and effectively detect epilepsy by Optimal Attention-Based Transformer-LSTM model, which significantly improves the interpretability and performance of the model. Yang et al. (15) used the Eurasian Oystercatcher Wild Geese Migration Optimization algorithm (EOWGMO) to optimize the feature weights to improve the fusion efficiency, and the Multiscale Dilated Adaptive DenseNet with Attention (MDADenseNet-AM) to obtain the converted text information, thus improving the performance of thought-to-text conversion. Ku et al. (16) applied LSTM to stock market forecasting, further demonstrating that LSTM can be applied to the forecasting function of time series. Okmi et al. (17) presents structured classification of data challenges and modeling strategies across high-dimensional temporal datasets, demonstrating the application of optimization strategies and deep learning to criminology. This paper proposes a Cox-Stuart-CNN-BiLSTM model for multi-patient data. The model is able to extract common characteristics of epileptic patients, and reduces time complexity, achieving an average test set accuracy of 0.9992, sensitivity of 0.9996, and specificity of 0.9988. For independent-patient data partitioning, propose an Optuna-CNN-BiLSTM model. This model effectively addresses the variability between different patient. The average test set accuracy, sensitivity, and specificity of the model are 0.9996, 0.9995, and 1.0000, respectively. The results indicate that the proposed models exhibit good predicted performance.

## 2 Materials and methods

### 2.1 CHB-MIT dataset

This study utilizes the CHB-MIT scalp EEG dataset of refractory epilepsy, collected by Boston Children's Hospital (18). The dataset comprises 23 subjects, with cases 1 and 21 belonging to the same patient, resulting in 24 folders. Data were collected using the International 10–20 system with a sampling rate of 256 Hz. The .edf format, specifically designed for recording EEG signals, was used. Each subject has between 9 and 42 continuous .edf files, documenting brain waveforms during both seizure and non-seizure periods, with seizure onset and offset times annotated by experts.

### 2.2 Preprocessing methods

#### 2.2.1 Seizure state segmentation

**Figure 1:**
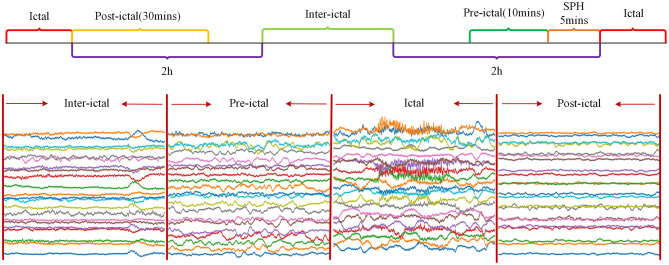
Illustration of epileptic state segmentation.

Since the CHB-MIT Epilepsy EEG dataset only labeled seizure start and end times, and not inter-ictal, pre-ictal, and post-ictal periods(post-ictal), it was decided in this chapter to define these periods manually. Seizure Prediction Horizon (SPH) is the period between the predicted occurrence of alarm and the start of seizure. In this chapter, the SPH was set to 5 min. The pre-ictal was defined as the period from 15 min before the seizure to the start of the SPH, which totalled 10 min. The post-ictal was defined as the period up to 30 min after the end of the seizure. The inter-ictal was defined as the period from 2 h after the end of the current seizure to 2 h before the start of the subsequent seizure. Figure 1 shows a schematic diagram of the division of status epilepticus. As can be seen from Figure 1, the waveform amplitude in the post-ictal showed the least change, and the waveform amplitude in the inter-ictal showed slight fluctuations. The waveform amplitude fluctuates more in the pre-ictal and the waveform amplitude fluctuates the most in the ictal.

#### 2.2.2 Dataset partitioning

Two data division strategies are used in this paper. The first strategy is a multi-patient data partitioning model, which is mainly applied to the case where the number of target patients is limited by integrating the data of other patients as the model training and validation sets. The second strategy is an independent patient data partitioning model, which is applicable to the case where the target patient have accumulated sufficiently data. This mode directly uses the data of target patient as the training set, validation set and test set of the model.

**Figure 2:**
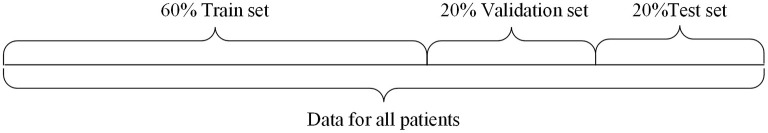
DIllustration of dataset partitioning based on muiti-patient.

Multi-patient data partitioning mode: The data of all patients are fully integrated, and then this dataset is partitioned into training, validation, and testing sets, which is suitable for the case of scarce data of target patient. In this paper, the multi-patient data division strategy is applied to Cox-Stuart-CNN-BiLSTM epilepsy EEG signal prediction model. The division is shown in Figure 2.

**Figure 3:**
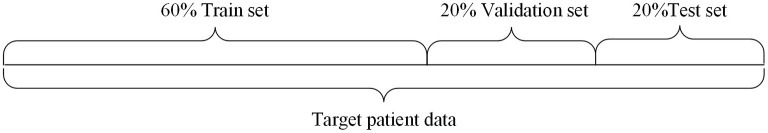
Illustration of dataset partitioning based on independent patient.

Independent patient data partitioning mode: The data of the target patient is partitioned into training, validation and testing sets, and then the corresponding data sets are put into the model for training, validation and testing, which is used in the case where the amount of data of the target patient is sufficient. In this paper, the data division method of independent patient is applied to the Optuna-CNN-BiLSTM epilepsy EEG signal prediction model. The division schematic is shown in Figure 3.

In this experiment, 20% of the data was randomly selected as the test set, while the remaining 80% was used for the training and validation sets. From this 80%, 20% was randomly chosen as the validation data. Thus, the proportions of the training, validation, and test sets are 6:2:2.

#### 2.2.3 Normalization

EEG signals in different seizure states may exhibit significant variations in amplitude, and the signal amplitudes across different EEG channels can also differ. To better distinguish between the inter-ictal and pre-ictal phases, it is crucial to normalize the raw data. In this study, the MinMaxScaler method from the Scikit-learn library was employed for standardization. MinMaxScaler linearly transforms the data to a specified range, as shown in Equation 1.


(1) $$\begin{array}{l} x_{scaled}=\left(x-x_{\min}\right)/\left(x_{\max}-x_{\min}\right)\times \left(\max-\min\right)+\min \end{array}$$

Here, *x* represents the raw data, *x*_*min*_ and *x*_*max*_ are the minimum and maximum values of the sequence, respectively, max and min denote the lower and upper bounds of the target scaling range. In this study, the default range is set to [0, 1], and *x*_*scaled*_ is the normalized data.

#### 2.2.4 Upsampling

Since the distribution of pre-ictal and inter-ictal EEG data in epilepsy patients is not balanced, for each patient, the seizure state data with less amount of data is slid in 10-s windows at an overlap rate of 50% to achieve the purpose of increasing the amount of data and balancing the sample size of seizure states. The number of windows n that the entire data series can be divided into is calculated by the following formula:


(2) $$\begin{array}{l} n=floor\left[\left(N-windows\times f_{s}\right)/overlop\times windows\right]+1 \end{array}$$

Where, the *floor* function indicates rounding down the data, *N* is the sequence length, which is set to 2,560, *f*_*s*_ is 256, and *overlop* is the overlap ratio, which is 50%.

#### 2.2.5 Filtering

**Figure 4:**
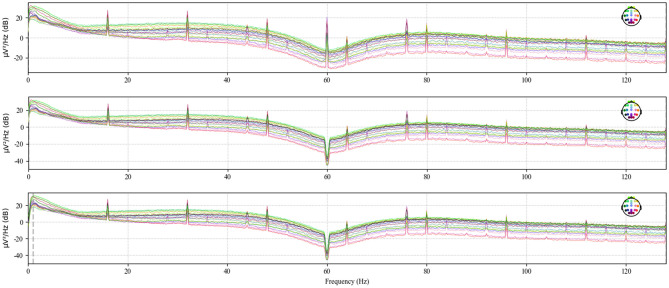
Power spectral density of chb01_01 after high-pass filtering.

Electromagnetic interference from the environment and thermal noise from the equipment can significantly disrupt the already weak EEG signals. To address power line interference noise, a notch filter at 50 or 60 Hz is typically used (19) In this study, the data from each .edf file was processed using the notch filter and high-pass filter functions from the mne library to eliminate 60 Hz power line noise and noise below 1 Hz, respectively (20). Figure 4 shows the original power spectral density, the power spectral density after notch filtering, and the power spectral density after high-pass filtering for the chb01_01 file.

### 2.3 Cox-Stuart early stopping mechanism

**Figure 5:**
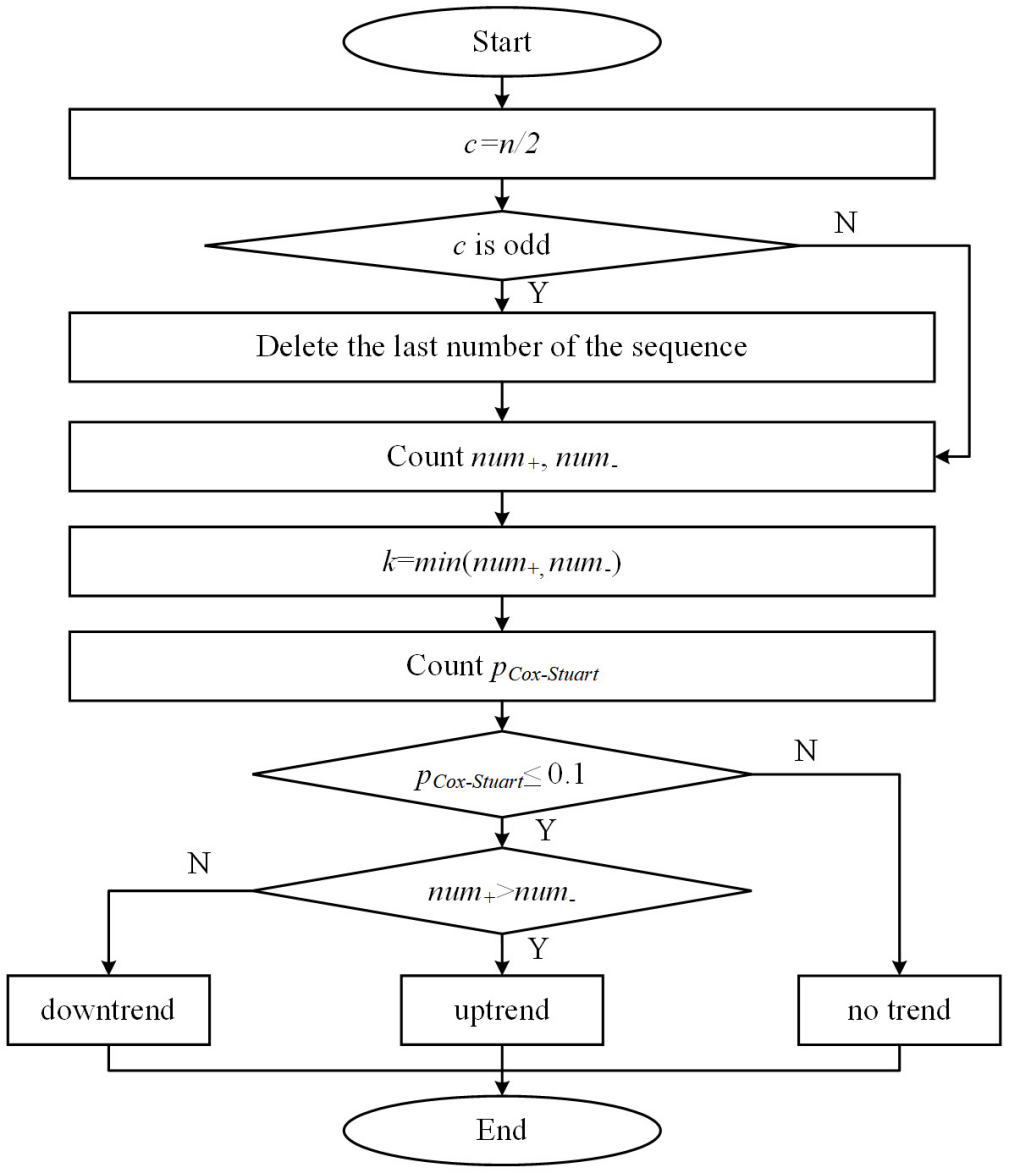
Flowchart of the Cox-Stuart test.

The Cox-Stuart test utilizes positive and negative signs to determine whether a sequence exhibits a certain trend. This method is applicable to various types of data and does not rely on data distribution, only on sign tests to identify upward or downward trends (21). The principle of the test can be understood as the data in the sequence is divided into two parts before and after, with the latter part of the sequence of values subtracted from the first half of the sequence of values to obtain several positive and negative differences in the sequence. The number of positive differences *num*_+_ is the number of differences >0. The number of negative differences *num*_−_ is the number of differences < 0. According to the hypothesis test, the original hypothesis *H*_0_ is considered to have no trend of change, and the alternative hypothesis *H*_1_ has a trend of change. When *num*_+_<*num*___, and at this time the probability of occurrence of positive difference *p*_*Cox*−*Stuart*_ ≤ 0.1, indicating that the sequence has a downward trend, rejecting *H*_0_; Similarly, when *num*_+_>*num*___ and at this time the probability of occurrence of negative difference *p*_*Cox*−*Stuart*_ ≤ 0.1, indicating that the sequence has an upward trend, rejecting the *H*_0_. If *num*_+_=*num*___, indicating that the sequence has no trend, accepting the *H*_0_. The specific calculation method is as follows. Figure 5 illustrates the flowchart of the Cox-Stuart test.

(1) Hypothesis: *H*_0_: The sequence has no trend; *H*_1_: The sequence exhibits a trend.
(2) Input the sequence {*x*_1_, *x*_2_, …*, x*_*n*_} and compute *c*, with the array length being *n*.

(3) $$c=\{\begin{array}{cc} n/2, & n\text{ is even}\\ (n+1)/2-1, & n\text{ is odd} \end{array}$$

(3) Calculate the paired values for set *c*: {*d*_1_ = *x*_*c*_ –* x*_0_*, d*_2_ = *x*_*c*+1_ –* x*_1_*,…, d*_*c*_ = *x*_*n*_ –* x*_*c*−1_}, count the number *num*_+_ and *num*___ in *c*, and set *k*=*min* (*num*_+_*, num*___).
(4) Utilize the cumulative probability function *p*_*Cox*−*Stuart*_ of the binomial distribution to compute the probability, where *p* represents the probability of observing either a single positive or negative sign.
(5) If *num*_+_>*num* and *p*_*Cox*−*Stuart*_ ≤ 0.1, the function is considered to have an uptrend. Conversely, if *num*_+_<*num*___ and *p*_*Cox*−*Stuart*_ ≤ 0.1, it is deemed to have a downtrend. Otherwise, no trend is observed.

### 2.4 Optuna optimization framework

The Optuna optimization framework was proposed in 2019 (22) which provides algorithms such as grid search method, stochastic search method, Covariance Matrix Adaptation Evolution Strategy (CMA-ES), and Tree-structured Parzen Estimator algorithm (TPE), which are able to adaptively find the optimal hyper-parameters of the model to optimize the objective function of the model and improve the performance.

The idea of the TPE algorithm is to use two different probability density functions *l*(*x*) and *g*(*x*) construct the conditional probability distribution of the model parameters: by constantly adjusting *l*(*x*) and *g*(*x*), the TPE algorithm is able to search the parameter space in a targeted way, from finding the global optimal solution. This is done as follows:

(1) Generates a random set of initial parameter configurations and evaluates their parameter performance.
(2) Updated conditional probability distribution.

(4) $$p(x|y)=\{\begin{array}{cc} l(x) & y<y^{*}\\ g(x) & y>y^{*} \end{array}$$

In the above equation, *x* is the input, *y* is the loss function, and y^*^ is the quartile of *y*. The specific quartile level is determined by the hyperparameter γ (which commonly takes the value of 0.15 or 0.25). Here, y^*^ is used as the target value threshold, and *p*(*x|y*) is split into the below-threshold conditional distribution *l*(*x*) and the above-threshold conditional distribution *g*(*x*), with *l*(*x*) denoting the loss function value lower than the target threshold, and *g*(*x*) indicating the probability density function when the loss function value is above the target threshold.
(3) Optimization parameter, $EI_{y^{*}}\left(x\right)$ is the expected improvement function.

(5) $$\begin{array}{l} EI_{y^{*}}\left(x\right)=\int_{-\infty }^{y^{*}}\left(y^{*}-y\right)\frac{p\left(x|y\right)p\left(y\right)}{p\left(x\right)} \end{array}$$

$$\begin{array}{l} dy=\frac{l\left(x\right)}{\gamma l\left(x\right)+\left(1-\gamma \right)g\left(x\right)}\int_{-\infty }^{y^{*}}\left(y^{*}-y\right)p\left(y\right)dy \end{array}$$

(4) Assessment parameter.

(6) $$\begin{array}{l} EI_{y^{*}}\left(x\right)\propto {\left(\gamma +\frac{g\left(x\right)}{l\left(x\right)}\left(1-\gamma \right)\right)}^{-1} \end{array}$$

It is easier to find the global optimal solution when the *g*(*x*) is minimum on *x* and the *l*(*x*) is maximum on *x*. The TPE algorithm has fewer iterations and quicker convergence, it is selected as the algorithm for the model.

This article also utilizes the Hyperband algorithm from the Optuna framework to promptly terminate experiments with poor training performance and reduce the training time.


(7) $$\begin{array}{l} s_{\max}=\log_{\eta }\left(\frac{R}{{\text{r}}_{\min}}\right) \end{array}$$

The above equation indicates that at most *s*_*max*_ evaluations can be performed. Where η denotes the proportion of parameters to be removed each time, *r*_*min*_ is the minimum resource, and *R* is the total resource. For a fixed η, *s*_*max*_ has different values. A larger *s*_*max*_ means smaller resources and a higher probability of early stopping, but there is a situation where the optimal solution cannot be found, on the contrary, smaller *s* means more enormous resources and a higher probability of finding the optimal solution, but it is unfavorable for early stopping. Based on this situation, the Hyperband algorithm tries all possible *s*, starting with the largest *s* until *s* = 0.

Compared to other optimization algorithms, Optuna offers the following advantages (23):

(1) Require minimal dependencies and can be used immediately after a simple installation, making it a lightweight, versatile, and cross-platform framework.
(2) Distributed optimization is straightforward.
(3) Allow for automatic early termination of hopeless experiments during the training phase, which reduces the time complexity of the model.

The steps for Optuna optimization are as follows:

(1) Define the search space: Determine the range of hyperparameters for Optuna to search within.
(2) Define the objective function: Optuna optimizes hyperparameters based on the objective function.
(3) Create an Optuna optimizer: Specify the objective function and search algorithm for the Optuna optimizer.
(4) Run the Optuna optimizer: Obtain the optimal hyperparameters by running the optimizer, train the model according to these hyperparameters, and return the objective function value. If a trial proves unpromising, it is automatically terminated, and the next trial continues.

### 2.5 Proposed model

**Figure 6:**
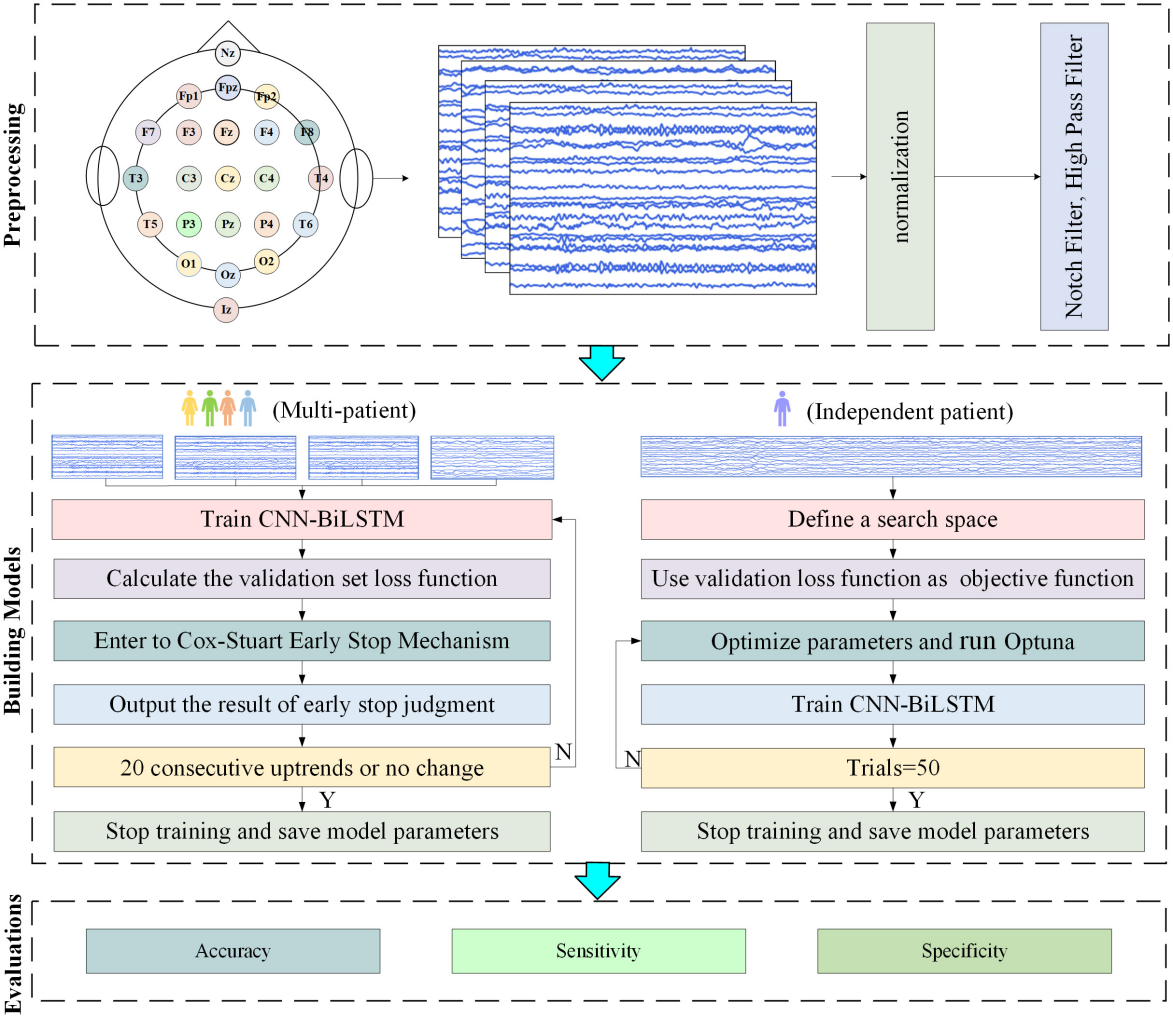
Flowchart of the experiment.

This article proposes a multi-patient and independent-patient epilepsy prediction model based on CNN-BiLSTM. The flowcharts of the two models are shown in Figure 6.

**Figure 7:**
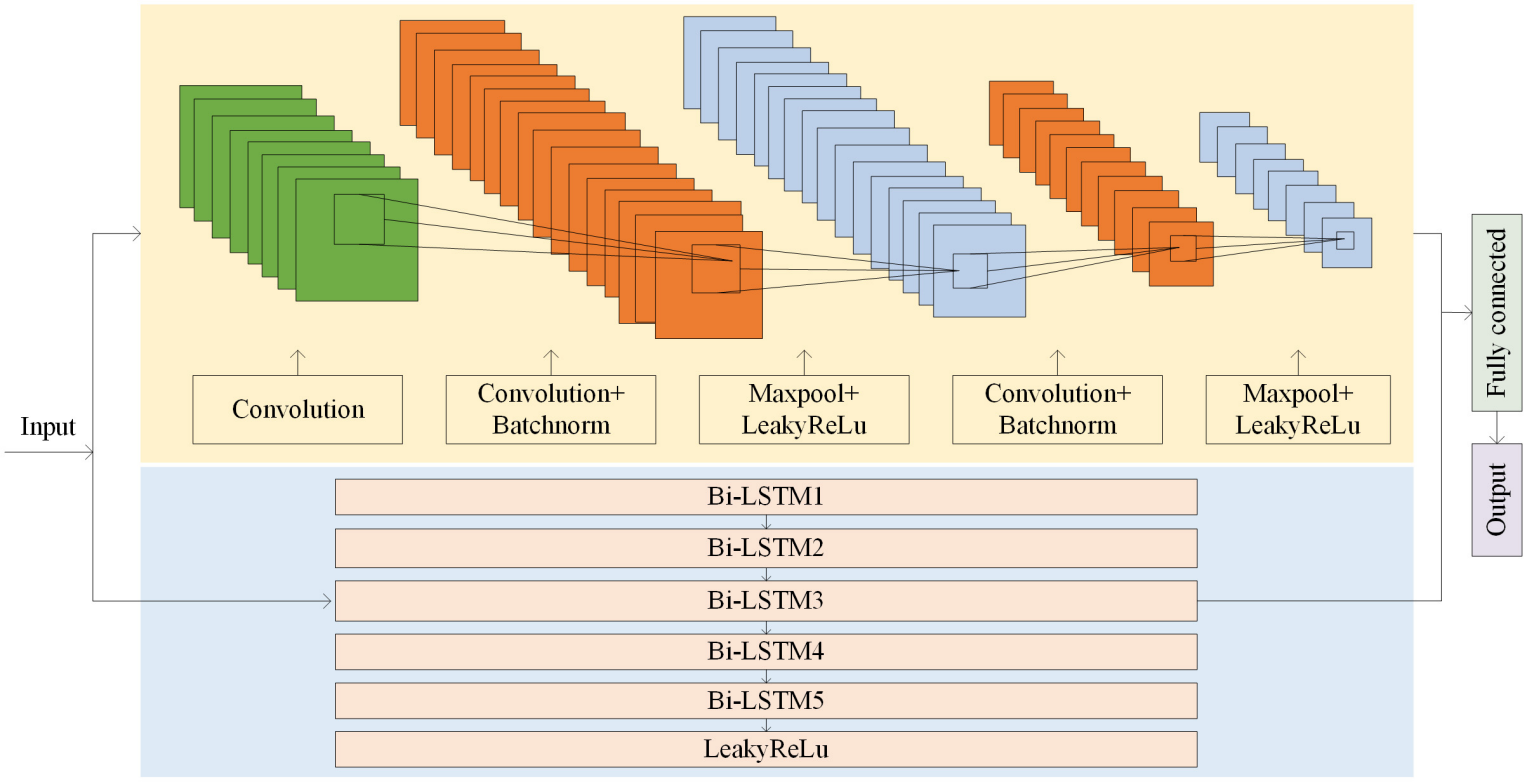
Model structure of the CNN-BiLSTM.

When the amount of target patient data is scarce, a model under a multi-patient data partitioning approach is proposed: a CNN-BiLSTM model based on the Cox-Stuart early stopping mechanism. The large amount of multi-patient data makes the CNN-BiLSTM model long in training time and high in computational complexity. To solve this problem, a Cox-Stuart early stopping mechanism is proposed to judge whether it is necessary to stop early according to the loss function of the validation set. The CNN-BiLSTM model structure is shown in Figure 7, and the model structure parameters are shown in Table 1. The model hyperparameters are shown in Table 2. The pseudo code is shown in Table 3.

**Table 1:** Model structure parameters.

**Layer**	**Input channels**	**Output channels**	**Convolutional kernel**	**Output dimension**	**Stride**
Input	22	–	–	(128,22,10,256)	–
2D convolution	22	22	1 × 1	(128,22,10,256)	1
2D convolution	22	44	3 × 3	(128, 44, 8, 254)	1
batch normalization	44	44	–	(128, 44, 8, 254)	
Maxpool	44	44	2 × 4	(128, 44, 4, 63)	1
Activation function	–	–	–	(128, 44, 4, 63)	
2D convolution	44	5	3 × 3	(128, 5, 2, 61)	1
batch normalization	5	5	–	(128, 5, 2, 61)	
Maxpool	5	5	2 × 4	(128, 5, 1, 15)	1
Activation function	–	–	–	(128, 5, 1, 15)	-
Bi-LSTM	2,560	1,280	–	(128, 22, 1,280)	1
Bi-LSTM	1,280	640	–	(128, 22, 640)	1
Bi-LSTM	640	160	–	(128, 22, 160)	1
Bi-LSTM	160	40	–	(128, 22, 40)	1
Bi-LSTM	40	2	–	(128, 22, 2)	1
Activation function	–	–	–	(128, 22, 2)	-
Fully connected	–	119	–	(128, 119)	-
Output	119	2	–	(128, 2)	-

**Table 2:** Hyperparameters of Cox-Stuart-CNN-BiLSTM model.

**Parameters**	**Parameter values**
Activation function	LeakyReLU
Loss function	CrossEntropyLoss
Optimizer	Adam
Learning rate	0.0001
Batch size	128
Epoch	Unsure
Weight_decay	0.9
Seed	42

**Table 3:** Pseudo code of Cox-Stuart-CNN-BiLSTM.

**Algorithm Cox-Stuart-CNN-BiLSTM model based on multi-patients**
Input:
D_train = {(x_*i*_, y_*i*_)}_{i=0, 1}, D_test = {(x_*j*_, y_*j*_)}_{j=0,1}
Model M(C,H,W), hyperparameters θ^*^, learning rateη, batch size B, epoch E, weight_decay λ, W_p
Output:
Cross-Validation metrics, test metrics, confusion matrices, spectrogram plots
01: set_seed(42)
02: init StratifiedKFold(n_splits=K, shuffle=True, seed=42)
03: best_states ← []; Cross-Validation metrics ← []
04: **for** fold = 1 to K do
05: split D_train → train_k, val_k
06: build DataLoader(train_k, B, shuffle=True) and DataLoader(val_k, B, shuffle=False)
07: M_k ← M().to(device)
08: optim ← Adam(M_k.params, η, weight_decay = λ)
09: sched ← ReduceLROnPlateau(optim, mode='min')
10: best_val_loss ←∞; c_inc = c_flat = 0; history ← empty lists
11: **for** e = 1 to E_max do
12: // Training loop
13: compute train_loss, train_acc, sens, spec over train_k
14: // Validation loop
15: compute val_loss, val_acc, sens, spec over val_k
16: sched.step(val_loss)
17: **if** val_loss < best_val_loss then
18: save_state(M_k); best_val_loss ← val_loss; reset(c_inc, c_flat)
19: **end if**
20: **if** epoch > W_p then
21: trend ← Cox_Stuart(last W_p val_losses)
22: **if** trend ε {“increasing”, “no_trend”} for W_p epochs then break
23: **end if**
24: **end for**
25: best_states.append(saved state)
26: record fold metrics; plot/save loss/acc/sens/spec curves
27: **end for**
28: // Select best across folds
29: θ^*^← state in best_states with minimal val_loss
30: // Test evaluation
31: compute cm_test on D_test; derive specificity, sensitivity, precision, accuracy
32: plot extended 3 × 3 confusion matrix; save figure
33: // Spectrograms
34: plot_test_samples(θ^*^, D_test, device, 5 preictal + 5 ictal)
35: **return** θ^*^, all recorded metrics, saved plots

The Optuna-CNN-BiLSTM model under the independent patient data division approach was used when the amount of collected target subject data was sufficient. Considering the significant differences in the amount of epileptic EEG data and the physiologic characteristics of patients, which may lead to large fluctuations in the experimental results of the CNN-BiLSTM model, the Optuna framework is introduced to model the hyperparameters for adaptive optimization. The hyperparameter settings of the model in this experiment are shown in Table 4. The pseudo code is shown in Table 5.

**Table 4:** Hyperparameters of Optuna-CNN-BiLSTM model.

**Parameters**	**Parameter values**
Activation function	LeakyReLU
Loss function	CrossEntropyLoss
Optimizer	Adam
Learning rate	[1e−5, 1e−2]
Batch size	[64, 128]
Epoch	[50, 200]
Weight_decay	[0.5, 1]
Trials	5
Seed	42

**Table 5:** Pseudo code of Optuna-CNN-BiLSTM.

**Algorithm Optuna-CNN-BiLSTM model based on independent patients**
Input:
• PATIENTS list, data_path, seed=42
• Model M(C,H,W)
• Search space:
– learning rate η ε [1e−5, 1e−2]
– batch size B ε {64,128}
– epochs E ε [50,200]
– weight decay λ ε [0.5,1.0]
• Metrics: accuracy, specificity, sensitivity
Output:
• Best model per patient, trial metrics, test confusion matrices, spectrograms, hyperparameter analysis plots
1: set_seed(42)
2: **for** each patient in PATIENTS do
3: load preictal/interictal .npy paths
4: split label → (x_train,y_train), (x_val,y_val), (x_test,y_test)
6: init train_ds, val_ds, train_loader, val_loader with default B
7: define objective(trial):
8: η←trial.suggest_float(‘lr',1e-5,1e-2)
9: B←trial.suggest_categorical(‘batch_size',[64,128])
10: E←trial.suggest_int(‘epoch',50,200)
11: λ←trial.suggest_loguniform(‘weight_decay',0.5,1.0)
12: model←M().to(device); optim←Adam(model.params,lr = η,wd = λ); loss_fn←CrossEntropyLoss
13: rebuild DataLoaders with B
14: best_val_loss←∞; best_state←None
15: **for** e=1 to E do
16: // Training
17: compute train_loss, acc, sen, spe over train_loader
18: // Validation
19: compute val_loss, acc, sen, spe over val_loader
20: **if** val_loss < best_val_loss then
21: best_val_loss←val_loss; best_state←model.state_dict()
22: **end if**
23: **end for**
24: save best_state to checkpoint_dir; record best metrics
25: return best_val_loss
26: **end** objective
27: study←Optuna.create_study(direction=‘minimize')
28: study.optimize(objective, n_trials=5)
29: best_trial←study.best_trial; print its params & value; compute avg±std across trials
30: load best_state; model←M().to(device); model.load_state_dict(best_state)
31: // Test evaluation
32: compute cm_test on x_test; derive specificity, sensitivity, precision, accuracy
33: plot extended 3 × 3 confusion matrix; save figure
34: // Spectrogram visualization
35: plot_test_samples(model, AudioDataset(x_test,y_test,…), device, 5 preictal, 5 ictal)
36: // Hyperparameter analysis
37: scatter lr, B, E, λ vs. best_val_loss; save plot
38: **end for**

### 2.6 Evaluation metrics

The evaluation metrics used are accuracy, sensitivity, and specificity. The accuracy refers to the probability of correct prediction among all predicted labels with the following equation.


(8) $$\begin{array}{l} accuracy=\frac{TP+TN}{TP+TN+FP+FN} \end{array}$$

Sensitivity refers to the probability that the model prediction will also be pre-ictal among all true pre-ictal labels, i.e., the accuracy of a positive result, with the following formula.


(9) $$\begin{array}{l} sensitivity=\frac{TP}{TP+FN} \end{array}$$

Specificity refers to the probability that the model prediction will also be inter-ictal in all true inter-ictal labeling, i.e., the accuracy of a negative result, with the following formula.


(10) $$\begin{array}{l} specificity=\frac{TN}{TN+FP} \end{array}$$

## 3 Results and discussion

### 3.1 Epilepsy prediction results based on multi-patient

#### 3.1.1 Epilepsy prediction model based on Cox-Stuart-CNN-BiLSTM

**Figure 8:**
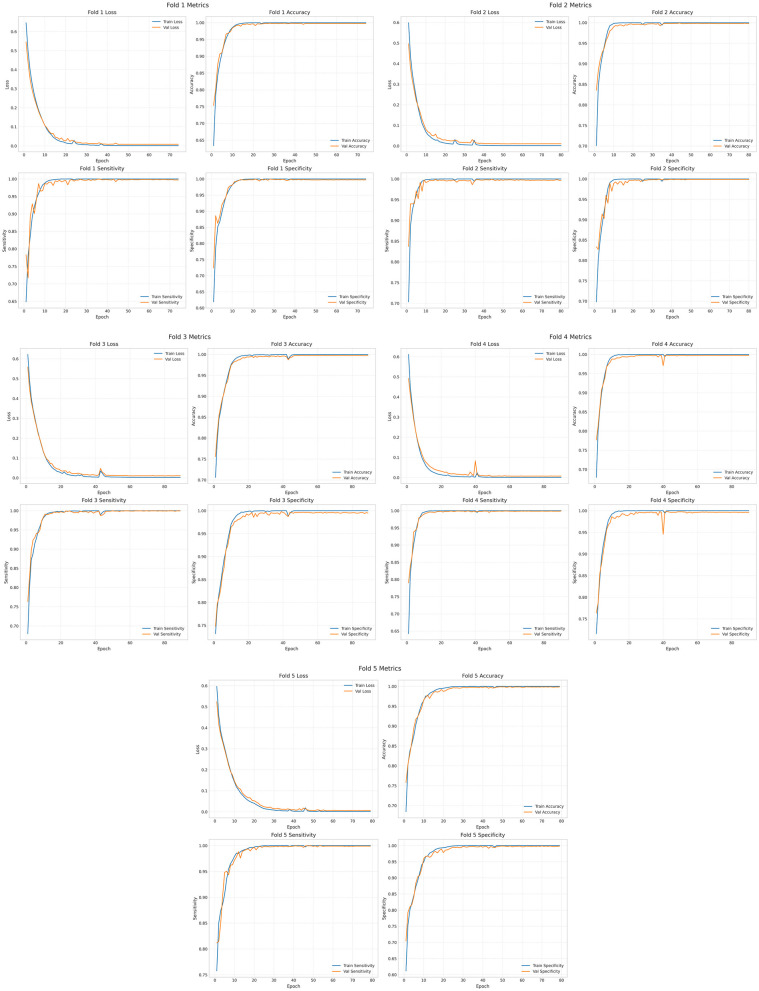
Training set and validation set of Cox-Stuart-CNN-BiLSTM.

The epoch is set to 250. The results of the training, validation and testing are shown in Figure 8 and Supplementary Figure 1. The loss values, accuracy, sensitivity and specificity of the five cross-validations are satisfactory. Supplementary Figure 2 shows the visualized time-frequency plots obtained by feeding the randomly selected test set data into the trained model, where True is the true label, True = 0 means pre-ictal, True = 1 means inter-ictal, and Pred is the prediction, Pred < 0.5 means the predicted label is 0, and Pred >0.5 means the predicted label is 1. It can be seen that the predicted results were all correct, and the inter-ictal phase has higher energy in the low frequency phase than the pre-ictal phase.

#### 3.1.2 Comparison of experimental results

In order to prove that the proposed model has obvious advantages, this paper compares CNN, CNN-BiLSTM, Threshold early stop-CNN-BiLSTM and the proposed model in this paper, and the results are shown in Table 6. Mean Val_Acc represents the average accuracy of the validation set, Mean Val_Sen represents the average sensitivity of the validation set, and Mean Val_Spe represents the average specificity of the validation set.Test_Acc denotes test set accuracy, Test_Sen denotes test set sensitivity, and Test_Spe denotes test set specificity. It can be seen that the cross-validation results and test set results of the four are not obvious in comparison, but compared with CNN, the Standard deviation (Std) is significantly lower after adding BiLSTM, which indicates that the model performance is more stable and the difference between the two in time is very small. The training time of the model is significantly shorter after adding the early-stop mechanism. Although the Cox-Stuart early stop has a long training time, its results are better than the threshold early stop.

**Table 6:** Comparison of the results of the four models.

**Evaluation metrics**	**CNN**	**CNN-BiLSTM**	**Threshold early stop-CNN-BiLSTM**	**Cox-Stuart-CNN-BiLSTM**
Mean Val_Acc±Std	0.9892 ± 0.003	0.9972 ± 0.0019	0.9968 ± 0.0020	0.9983 ± 0.0005
Mean Val_Sen±Std	0.99 ± 0.0032	0.9972 ± 0.0024	0.9967 ± 0.0025	0.9987 ± 0.0007
Mean Val_Spe±Std	0.9884 ± 0.0029	0.9972 ± 0.0017	0.9969 ± 0.0016	0.9979 ± 0.0010
Test_Acc	0.9939	0.9988	0.9986	0.9992
Test_Sen	0.9921	0.9992	0.9984	0.9996
Test_Spe	0.9957	0.9984	0.9988	0.9988
Train Time(s)	9,246	9,316	1,867	2,990

In order to further validate the performance of the proposed model, this paper uses *t*-test and Cohen's *d* to statistically analyze the proposed model and other models, when the *p*-value is < 0.05, it means that the two models are significantly different, otherwise, it means that there is no significant difference in the results. However, focusing only on the *p*-value has some limitations, therefore, this paper also used Cohen's *d* to verify the model differences (24), When |Cohen's *d*| < 0.20 proves that the difference between the two models is insignificant, 0.2 < |Cohen's *d*| < 0.50 proves that there is a small difference between the two models, 0.5 < |Cohen's *d*| < 0.80 proves that there is a medium difference between the two models, and when | Cohen's *d*| > 0.80 proves that there is a significant difference between the two models (25) The results are shown in Table 7, and it can be seen that the results of Cox-Stuart-CNN-BiLSTM outperform the CNN, outperform the results of Threshold early stop-CNN-BiLSTM, and are not significantly different from the results of CNN-BiLSTM.

**Table 7:** Significant difference results with Cox-Stuart-CNN-BiLSTM.

**Evaluation metrics**	**CNN vs. Cox-Stuart-CNN-BiLSTM**		**CNN-BiLSTM vs. Cox-Stuart-CNN-BiLSTM**		**Early stop-CNN-BiLSTM vs. Cox-Stuart-CNN-BiLSTM**	
	***p***	**| Cohen's *d* |**	***p***	**| Cohen's *d* |**	***p***	**| Cohen's *d* |**
Mean Val_Acc	0.0021	4.2314	0.2711	0.7918	0.1711	1.029
Mean Val_Sen	0.0030	3.7561	0.2412	0.8485	0.1503	1.0895
Mean Val_Spe	0.0010	4.3797	0.4555	0.5019	0.2762	0.7495

Table 8 and Supplementary Figure 3 show the experimental results of the Cox-Stuart-CNN-BiLSTM model compared with other models proposed in the literature. As shown in Table 8 and Supplementary Figure 2, this study achieves the highest accuracy, sensitivity, and specificity among the six, even with only data filtering applied. Additionally, this study employs an early stopping mechanism that adaptively adjusts the epoch size, reducing time complexity.

**Table 8:** Comparison of experimental results with other models.

**Literatures**	**Data set**	**Preprocessing**	**Model**	**Test_Acc**	**Test_Sen**	**Test_Spe**
(27)	CHB-MIT	EMD	CNN	0.9978	–	–
(28)	CHB-MIT	Notch, Bandpass	CDAN	0.7090	–	–
(29)	CHB-MIT	WT	1D-CNN	0.8650	0.8440	–
(29)	Non-public	3D image	3D-CNN	0.9237	0.8890	0.9378
(30)	CHB-MIT	Highpass, Lowpass, Notch	CNN	0.8217	0.8580	0.7402
This paper	CHB-MIT	Notch Filter, High Pass Filter	Cox-Stuart- CNN-BiLSTM	0.9992	0.9996	0.9988

### 3.2 Epilepsy prediction results based on independent patient

#### 3.2.1 Optuna-CNN-BiLSTM epilepsy prediction model

The independent patient-based Optuna-CNN-BiLSTM model is able to train personalized model parameters based on the patient's own conditions and further improve the interpretability of the model parameters. Table 9 shows the best model hyperparameters for 23 subjects, which have an average learning rate of 0.0061, an average Batch size of 109, an average epoch of 134, and an average weight_decay is 0.7007.

**Table 9:** Best hyperparameters for the 23 subjects.

**Subjects**	**Best hyperparameters**			
	**Learning rate**	**Batch size**	**Epoch**	**Weight_decay**
chb01	0.0073	128	184	0.5467
chb02	0.0027	64	171	0.6191
chb03	0.0015	64	130	0.5986
chb04	0.0100	128	133	0.8397
chb05	0.0053	128	127	0.7894
chb06	0.0037	64	115	0.5150
chb07	0.0085	128	159	0.8994
chb08	0.007	128	168	0.7281
chb09	0.0091	64	104	0.5555
chb10	0.0011	128	126	0.5095
chb11	0.0074	128	137	0.6919
chb12	0.0055	128	187	0.6217
chb13	0.0033	128	105	0.7723
chb14	0.0057	64	61	0.8377
chb15	0.0002	128	111	0.9027
chb16	0.0097	128	165	0.7252
chb17	0.0097	128	110	0.8361
chb18	0.0036	128	104	0.6273
chb19	0.0092	64	144	0.5959
chb20	0.0088	128	136	0.9236
chb21	0.0079	64	153	0.6968
chb22	0.0060	128	119	0.6222
chb23	0.0077	128	122	0.6613
Mean	0.0061	109	134	0.7007

Supplementary Figures 4, 5 show the visualization of the best hyperparameters for the 23 subjects. Given Trials = 5, each subplot comprises five discrete points. The first subplot illustrates the relationship between learning rate and optimal validation loss, showing that loss decreases as learning rate increases but rises again when rates become excessively high due to unstable, overly large parameter updates. The second subplot depicts batch size vs. optimal validation loss, demonstrating that optimal batch size varies across subjects and that selecting it appropriately balances gradient variance with computational efficiency. The third subplot shows that validation loss decreases with the number of training epochs, converging by approximately 160 epochs and yielding negligible improvement when extended to 200 epochs. Finally, the fourth subplot illustrates the effect of weight decay on validation loss: increasing weight decay from 0.5 to 0.75 reduces loss to a minimum at 0.75, while a further increase to 1.0 induces underfitting via strong L2 regularization and causes loss to rise.

After Trials = 5 times of training, the results of the validation set and test set are obtained as shown in Table 10, which shows that the Accuracy of the Validation set(Val_Acc), Sensitivity of the Validation set(Val_Spe), and Specificity of the Validation set(Val_Spe) are above 0.98, the mean values are above 0.99, and the Std are all around 0.01, indicating the model stability. Supplementary Figures 6, 7 show the test set confusion matrix for the 23 subjects.

**Table 10:** Validation set and test set results for 23 subjects.

**Subjects**	**Val_Acc±Std**	**Val_Sen±Std**	**Val_Spe±Std**	**Train time**	**Data volume**
chb01	1 ± 0.0000	1 ± 0.0000	1 ± 0.0000	1,509	1,328
chb02	0.9968 ± 0.0063	1 ± 0.0000	0.9937 ± 0.0126	1,319	476
chb03	0.9912 ± 0.0072	0.9824 ± 0.0144	1 ± 0.0000	1,498	1,194
chb04	1 ± 0.0000	1 ± 0.0000	1 ± 0.0000	1,365	714
chb05	0.9979 ± 0.0042	0.9966 ± 0.0068	0.9992 ± 0.0017	1,246	1,184
chb06	1 ± 0.0000	1 ± 0.0000	1 ± 0.0000	1,286	1,650
chb07	1 ± 0.0000	1 ± 0.0000	1 ± 0.0000	1,377	714
chb08	1 ± 0.0000	1 ± 0.0000	1 ± 0.0000	1,480	1,190
chb09	1 ± 0.0000	1 ± 0.0000	1 ± 0.0000	1,232	714
chb10	1 ± 0.0000	1 ± 0.0000	1 ± 0.0000	1,377	1,666
chb11	1 ± 0.0000	1 ± 0.0000	1 ± 0.0000	1,038	476
chb12	0.9932 ± 0.0084	0.9864 ± 0.0167	1 ± 0.0000	1,072	880
chb13	1 ± 0.0000	1 ± 0.0000	1 ± 0.0000	1,041	1,230
chb14	1 ± 0.0000	1 ± 0.0000	1 ± 0.0000	1,291	1,428
chb15	1 ± 0.0000	1 ± 0.0000	1 ± 0.0000	1,641	2,982
chb16	0.9994 ± 0.0012	1 ± 0.0000	0.9988 ± 0.0024	1,556	842
chb17	1 ± 0.0000	1 ± 0.0000	1 ± 0.0000	1,171	714
chb18	1 ± 0.0000	1 ± 0.0000	1 ± 0.0000	1,161	1,014
chb19	1 ± 0.0000	1 ± 0.0000	1 ± 0.0000	1,438	476
chb20	1 ± 0.0000	1 ± 0.0000	1 ± 0.0000	1,625	1,190
chb21	1 ± 0.0000	1 ± 0.0000	1 ± 0.0000	1,553	952
chb22	1 ± 0.0000	1 ± 0.0000	1 ± 0.0000	1,564	714
chb23	0.9977 ± 0.0045	0.9955 ± 0.0091	1 ± 0.0000	1,111	660
Mean	0.9989 ± 0.0014	0.9983 ± 0.0020	0.9996 ± 0.0007	1,346	1,060

Supplementary Figures 8–11 are visualization of the time-frequency plots obtained by feeding data from a randomly selected test set of 23 subjects into the trained model, and it can be seen that the predictions are all correct and that the inter-ictal phase is more energetic than the pre-ictal phase in the low-frequency phase.

#### 3.2.2 Comparative experiments

Table 11 shows the average accuracy, average sensitivity, and average specificity of the test set of CNN, CNN-BiLSTM, and Optuna-CNN-BiLSTM models. It can be seen that the addition of BiLSTM improves the model results. Although the difference between the results of CNN-BiLSTM and Optuna-CNN-BiLSTM is small and the difference in training time is large, Optuna provides interpretability for the model parameters and the model performance is more stable, which is beneficial for subsequent research.

**Table 11:** Comparison of the results of the three models.

**Model**	**Test_Acc**	**Test_Sen**	**Test_Spe**	**Train time(s)**
CNN	0.9984	0.9972	0.9993	435
CNN-BiLSTM	0.9996	0.9991	1	453
Optuna-CNN-BiLSTM	0.9996	0.9995	1	1346

Table 12 and Supplementary Figure 12 show the experimental results of the Optuna-CNN-BiLSTM model compared with other models proposed in the literature.Under the same data set, the proposed model has the highest accuracy, sensitivity and specificity compared with other models in the literature. It significantly proves that the Optuna-CNN-BiLSTM epilepsy EEG signal prediction model has excellent performance.

**Table 12:** Comparison of experimental results with other models.

**Literatures**	**Data set**	**Preprocessing**	**Model**	**Test_Acc**	**Test_Sen**	**Test_Spe**
(31)	CHB-MIT	–	Lightweight-2D-CNN	0.8998	0.9290	0.8704
(9)		–	NSGA-II+CNN	0.9651	0.9655	0.9647
(32)		–	CNN-SVM	0.8625	–	–
(11)		Filter, STFT	CNN-LSTM	0.9400	0.938	0.912
(13)		–	CGAN- TACNN-LSTM	0.946	0.945	–
(12)		–	CNN-BiLSTM	0.9483	0.9494	–
This paper		Filter	Optuna-CNN-BiLSTM	0.9996	0.9995	1

## 4 Conclusion

This paper focuses on the preprocessing of epileptic EEG signals, the construction of an epileptic EEG signal prediction model based on deep learning and its performance analysis. A Cox-Stuart-CNN-BiLSTM epilepsy EEG signal prediction model based on multiple patients is proposed for the case of scarce data of target patients, which achieves 0.9992 accuracy, 0.9996 sensitivity and 0.9988 specificity. For the case of sufficient data of target patients, an independent patient-based Optuna-CNN- BiLSTM epilepsy EEG signal prediction model is proposed, which achieved 0.9996 accuracy, 0.9995 sensitivity and 1.0000 specificity. The epilepsy prediction model can provide a timely warning of epileptic seizures and help patients take preventive measures in advance, thus reducing the harm caused by epileptic seizures to the physical and mental health of patients. Although this paper has made great progress in epilepsy prediction, there are still the following shortcomings: (1) The experimental data are too monotonous. This paper uses data from patients with refractory epilepsy. Although the experimental results are great, the effect on EEG data of other epilepsy types is not yet known. Additional epilepsy prediction data will be added in the future to further highlight the generalization ability of the model. (2) The interpretability of the model parameters needs to be improved, and in the future we will refer to the method proposed by He et al. (14) to improve the interpretability of the model parameters through the parameter selection strategy. (3) Noise that exists in the real world is more complex and will be added to analog noise in the future (26), in order to be more in tune with reality. (4) Attention mechanism will be added in the future to further support the model performance.

## Data Availability

Publicly available datasets were analyzed in this study. This data can be found here: https://physionet.org/content/chbmit/1.0.0/#files-panel.

## Supplementary material

Supplementary Figure 1 Test set results of Cox-Stuart-CNN-BiLSTM.

Supplementary Figure 2 Time-frequency plot of test set visualization.

Supplementary Figure 3 Experimental results of different models.

Supplementary Figure 4 Visualization of best hyperparameters for subjects 1–12.

Supplementary Figure 5 Visualization of best hyperparameters for subjects 13–23.

Supplementary Figure 6 Test set confusion matrix for subjects 1–12.

Supplementary Figure 7 Test set confusion matrix for subjects 13–23.

Supplementary Figure 8 Time-frequency plots of test set visualizations for subjects 1–6.

Supplementary Figure 9 Time-frequency plots of test set visualizations for subjects 7–12.

Supplementary Figure 10 Time-frequency plots of test set visualizations for subjects 12–18.

Supplementary Figure 11 Time-frequency plots of test set visualizations for subjects 19–23.

Supplementary Figure 12 Experimental results of different models.
