# Supplementary figures and images for: A novel epileptic seizure prediction model based on Cox-Stuart and Optuna
Source: Front Neurol. 2025 Oct 16;16:1624873. doi: 10.3389/fneur.2025.1624873 (PMC12621226; doi:10.3389/fneur.2025.1624873)

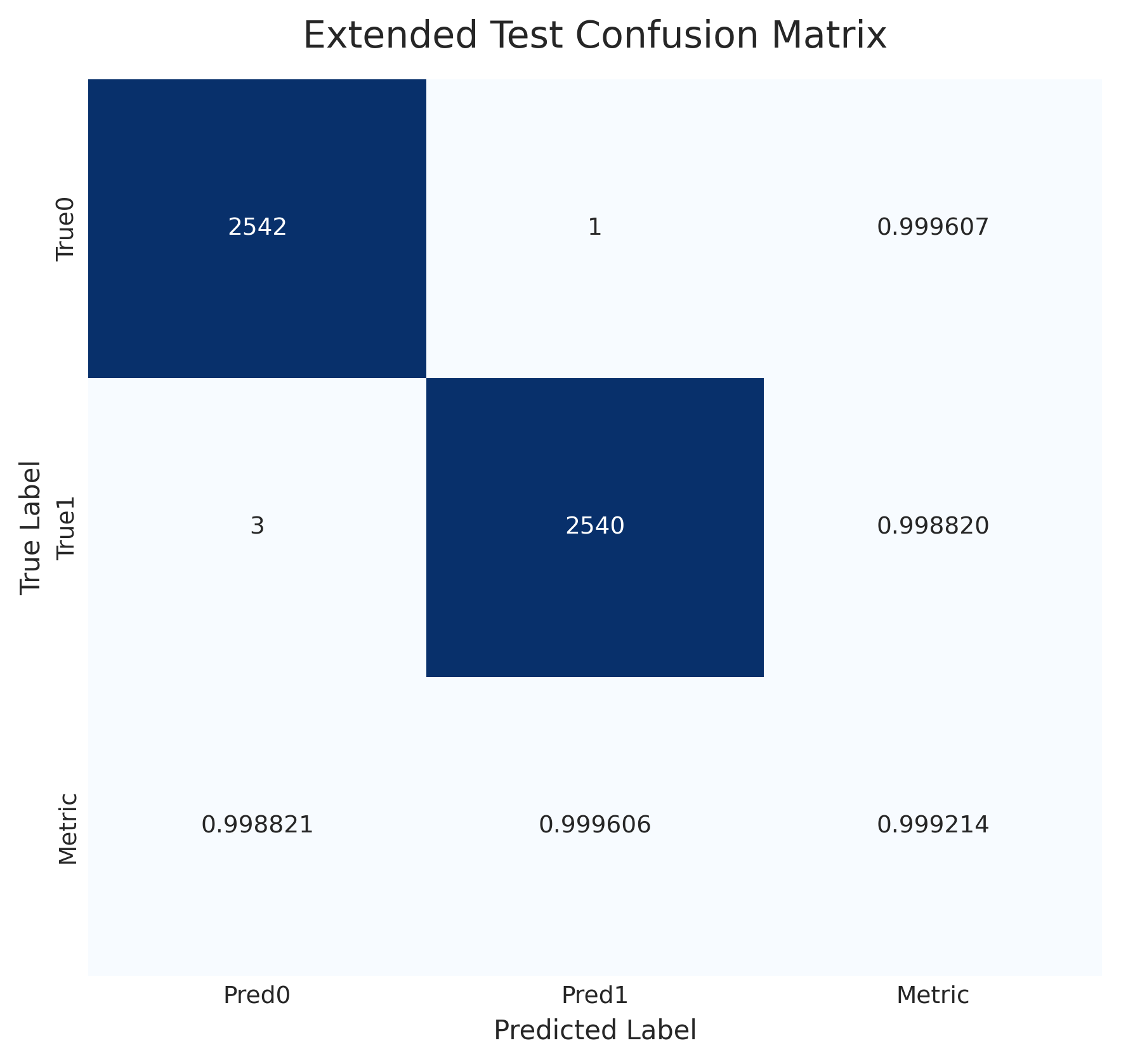

Supplement: Supplementary Figure 1 — Test set results of Cox-Stuart-CNN-BiLSTM. [file Image_1.png]

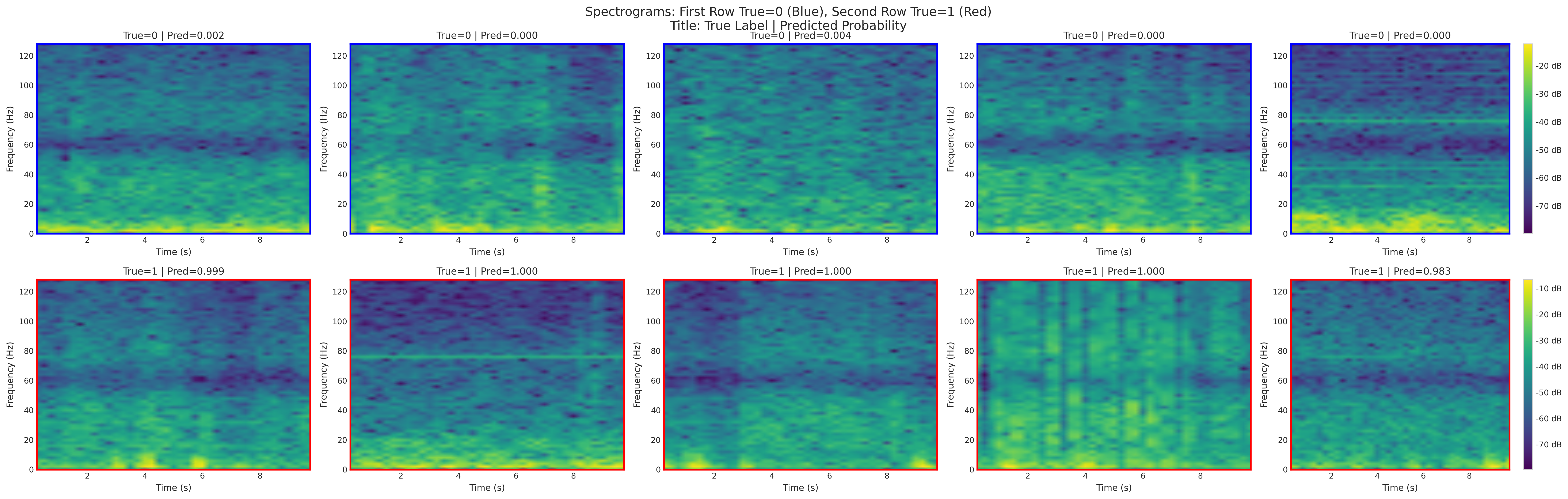

Supplement: Supplementary Figure 2 — Time-frequency plot of test set visualization. [file Image_2.png]

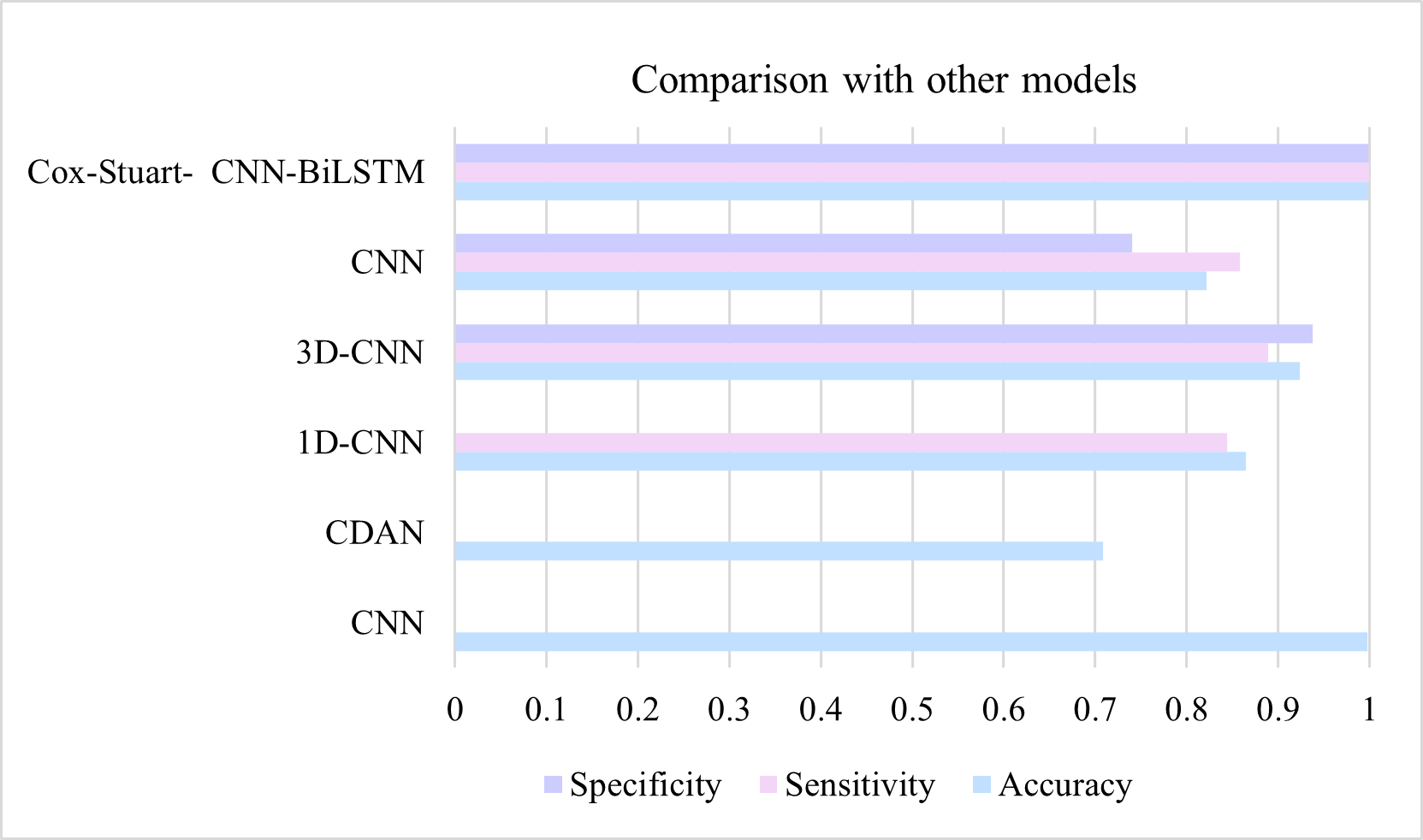

Supplement: Supplementary Figure 3 — Experimental results of different models. [file Image_3.tif]

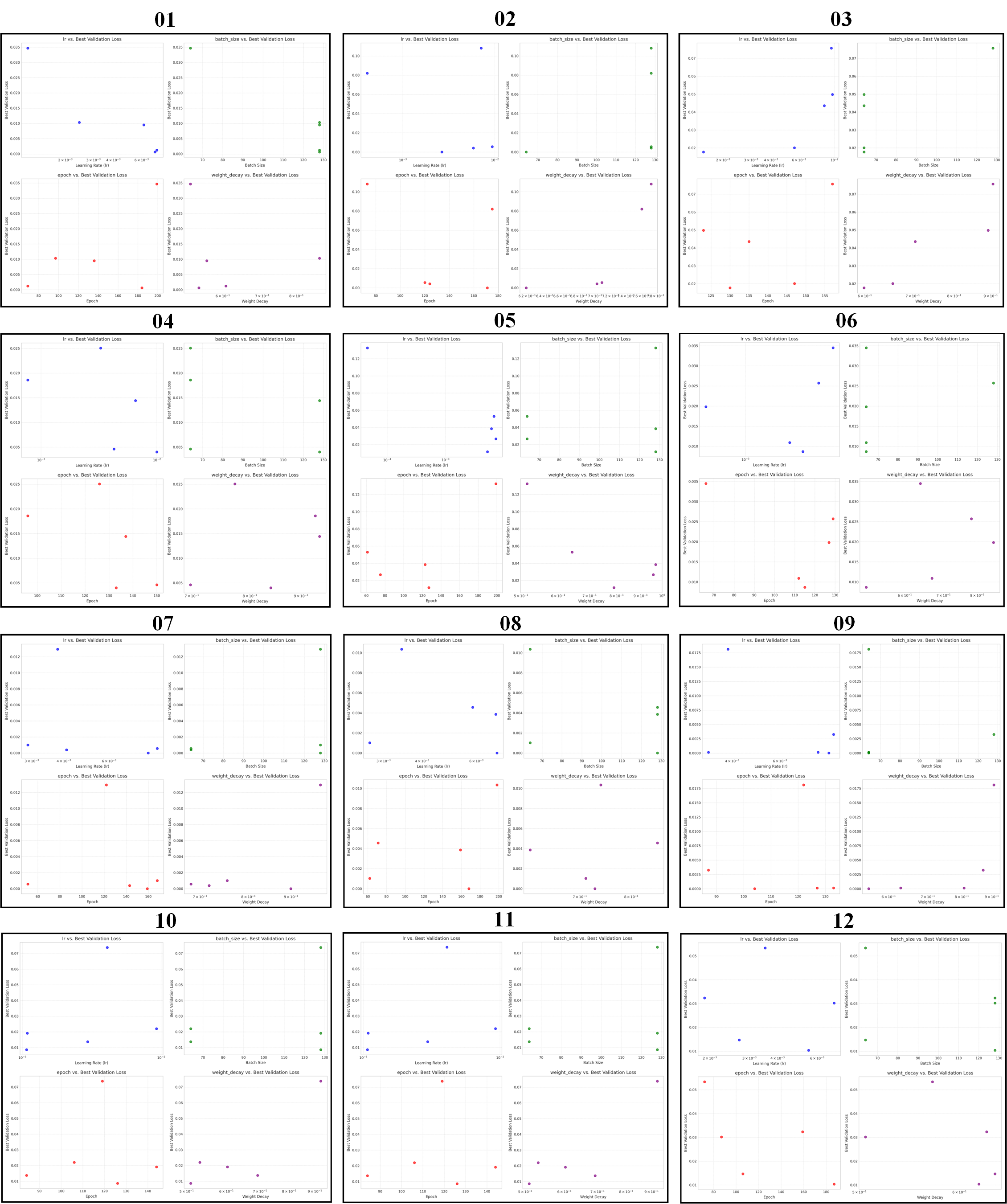

Supplement: Supplementary Figure 4 — Visualization of best hyperparameters for subjects 1–12. [file Image_4.tif]

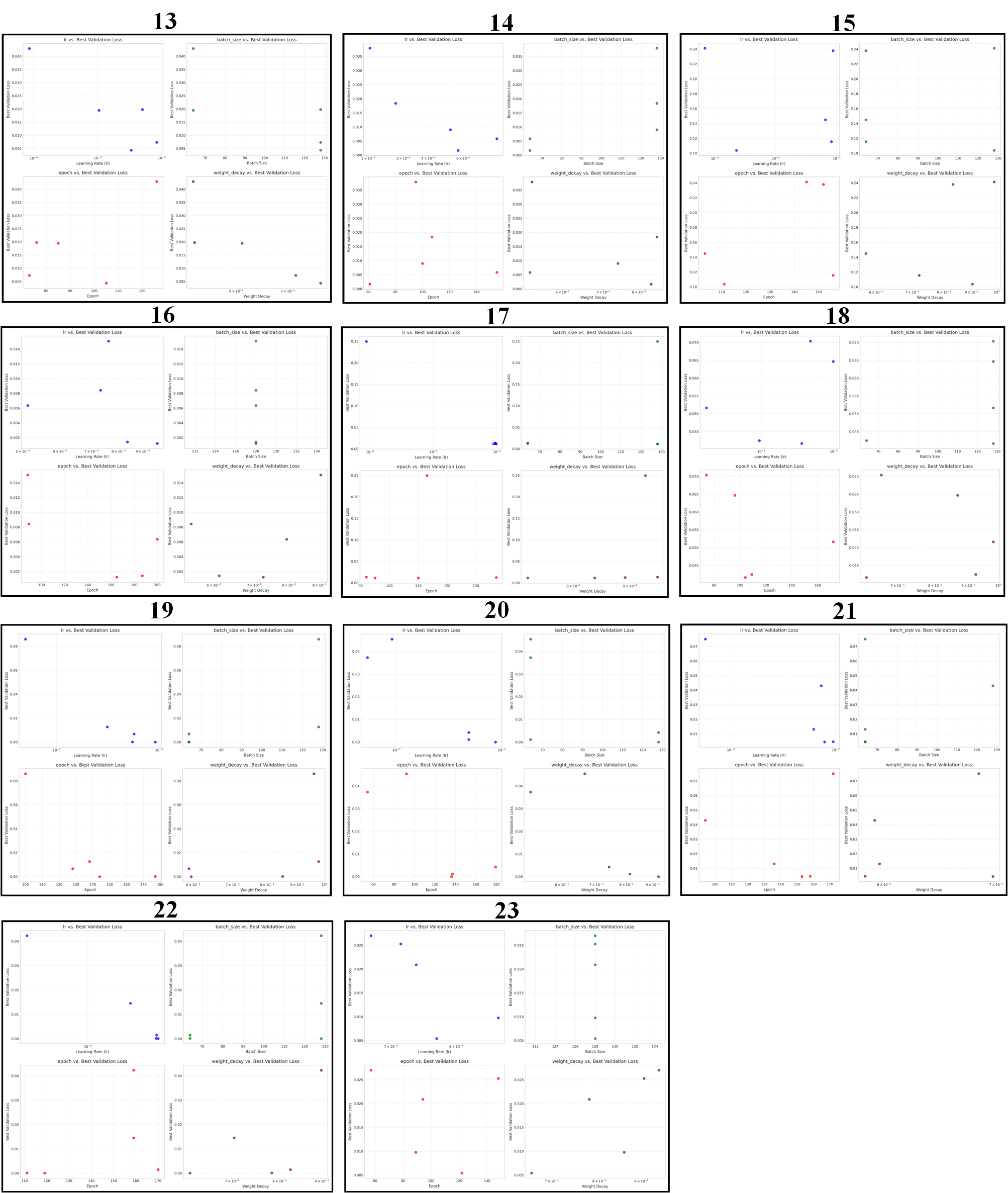

Supplement: Supplementary Figure 5 — Visualization of best hyperparameters for subjects 13–23. [file Image_5.tif]

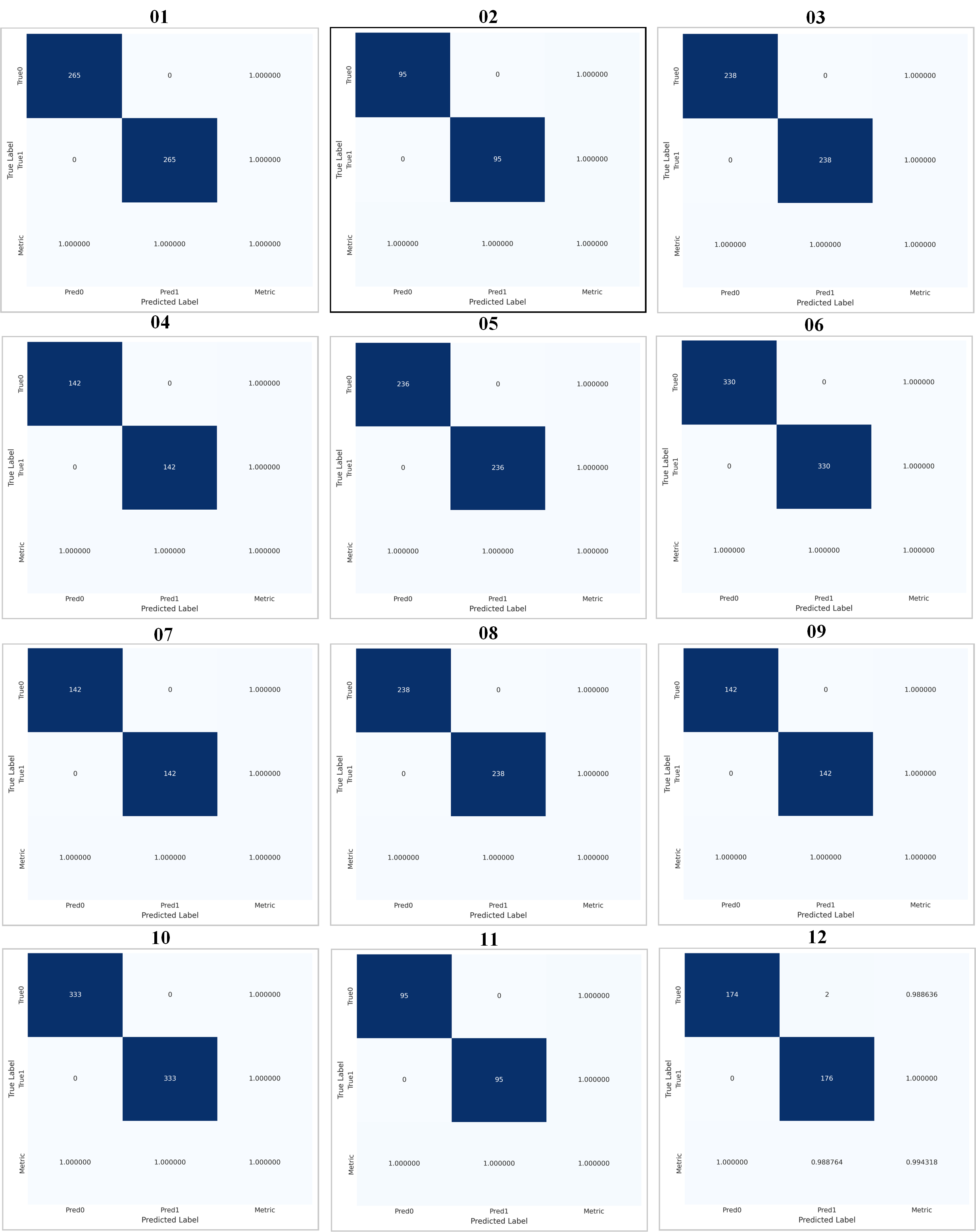

Supplement: Supplementary Figure 6 — Test set confusion matrix for subjects 1–12. [file Image_6.tif]

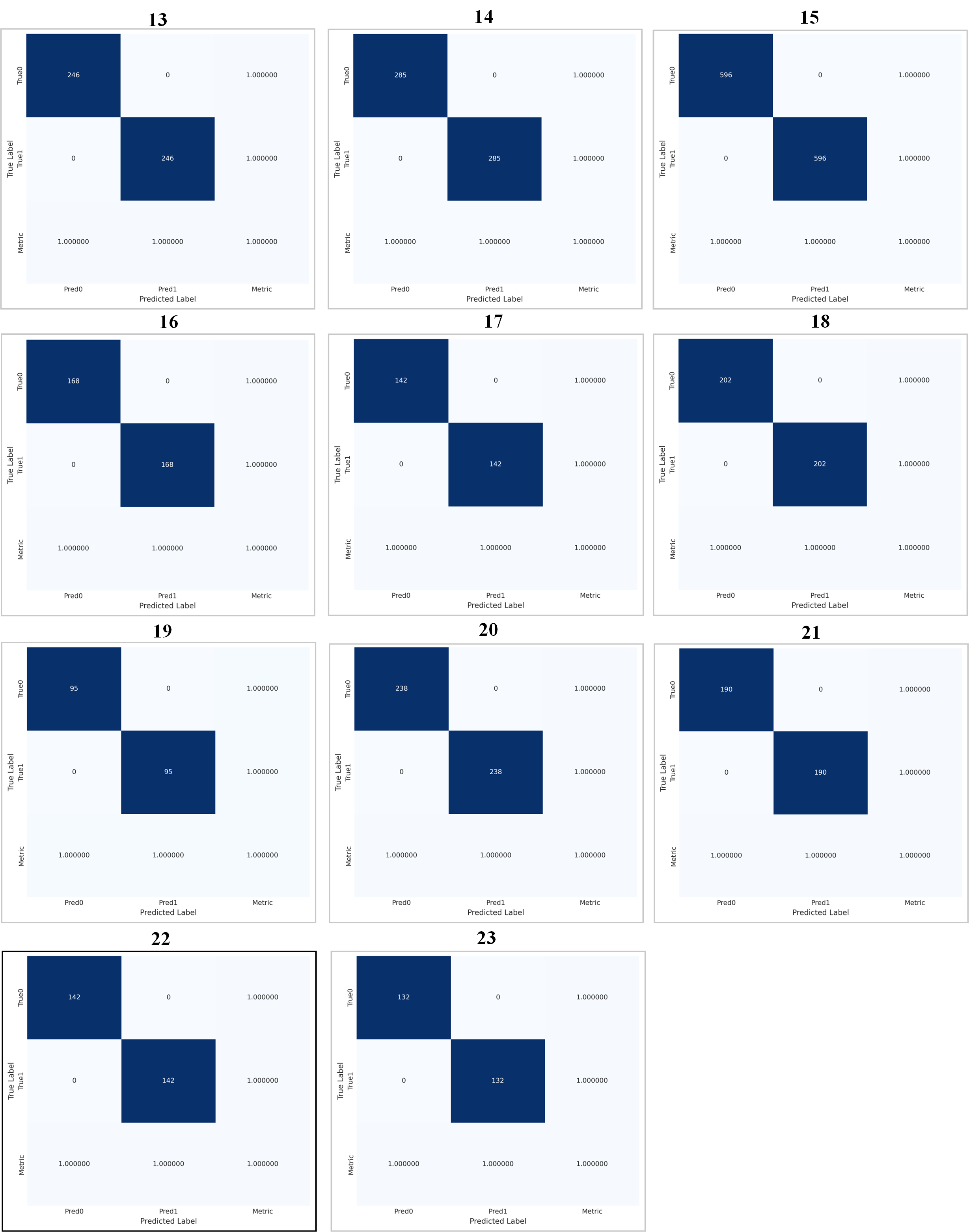

Supplement: Supplementary Figure 7 — Test set confusion matrix for subjects 13–23. [file Image_7.tif]

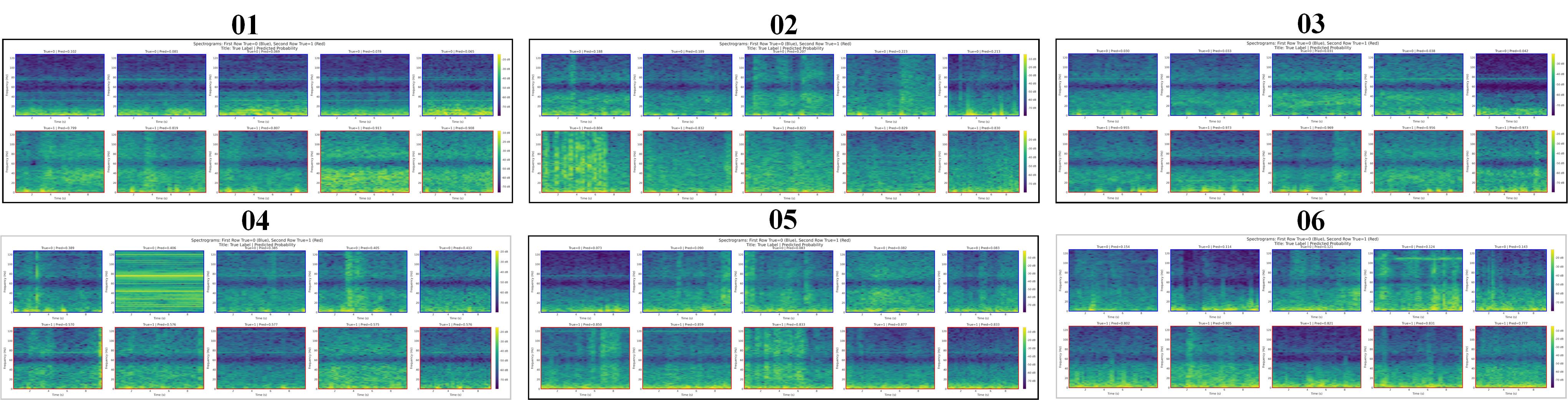

Supplement: Supplementary Figure 8 — Time-frequency plots of test set visualizations for subjects 1–6. [file Image_8.tif]

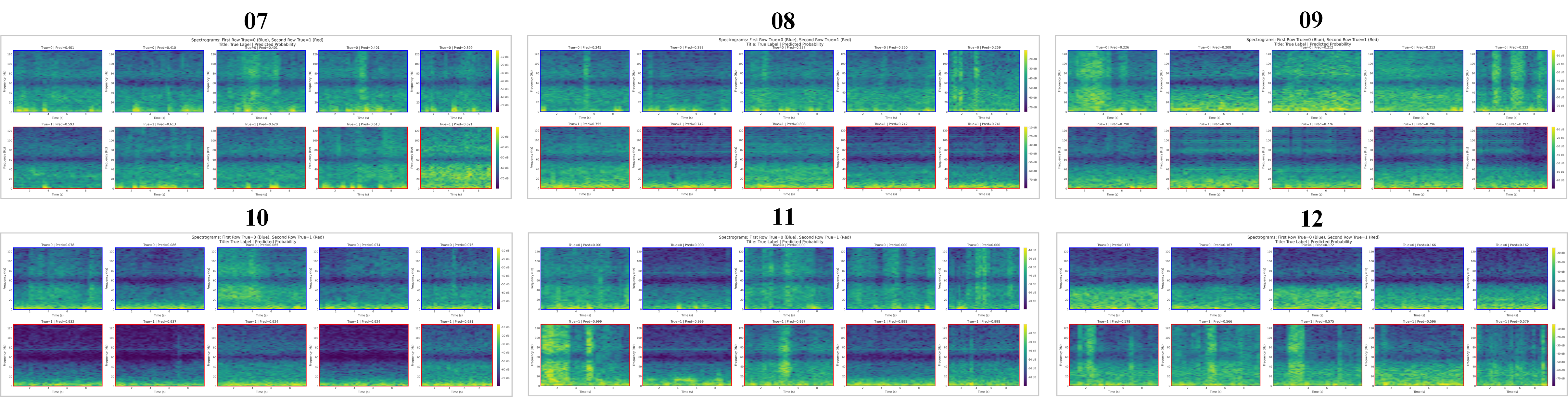

Supplement: Supplementary Figure 9 — Time-frequency plots of test set visualizations for subjects 7–12. [file Image_9.tif]

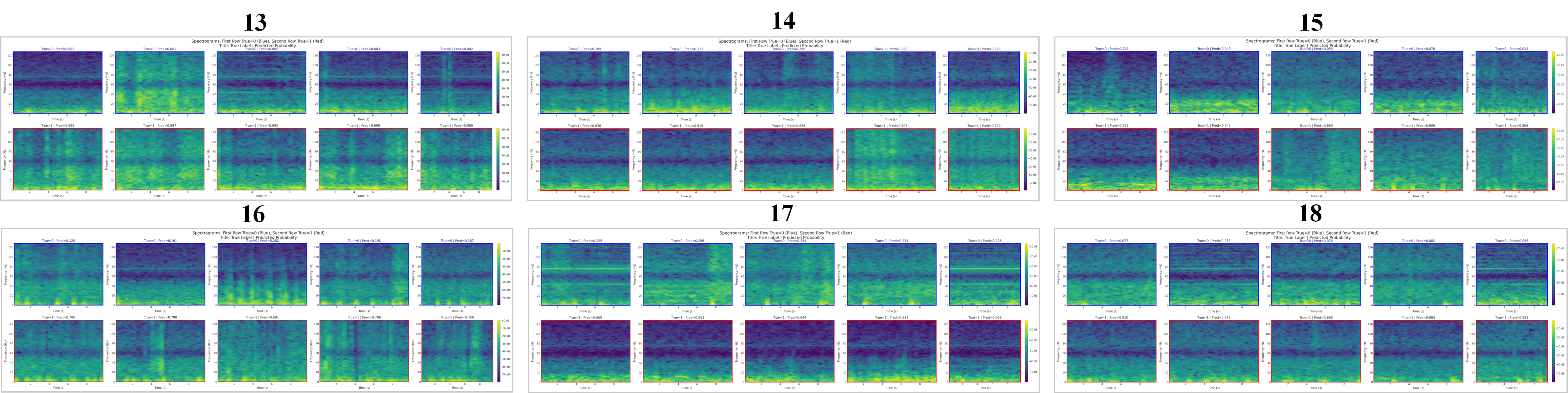

Supplement: Supplementary Figure 10 — Time-frequency plots of test set visualizations for subjects 12–18. [file Image_10.tif]

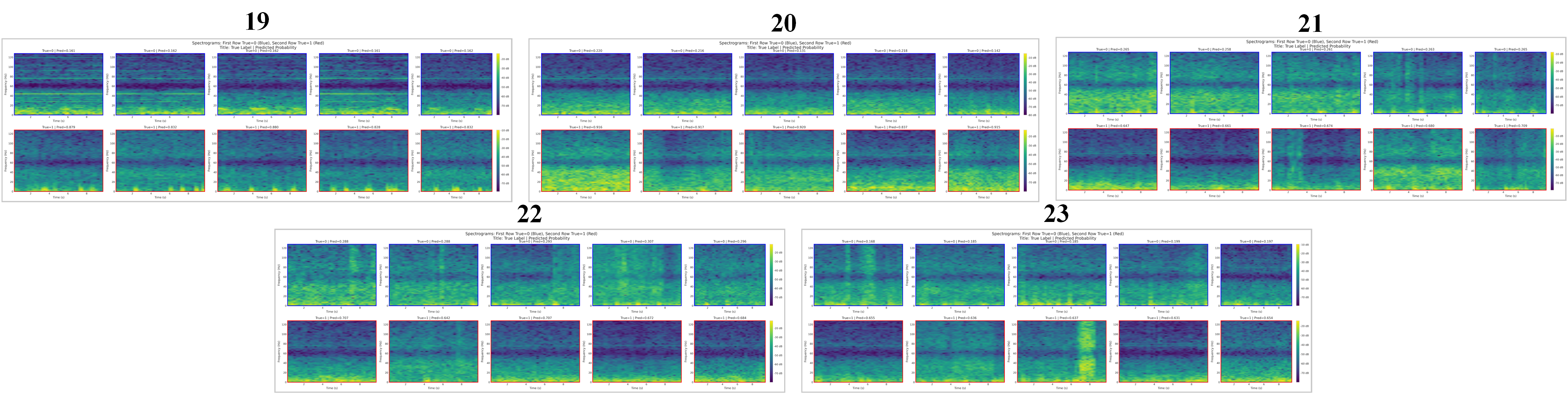

Supplement: Supplementary Figure 11 — Time-frequency plots of test set visualizations for subjects 19–23. [file Image_11.tif]

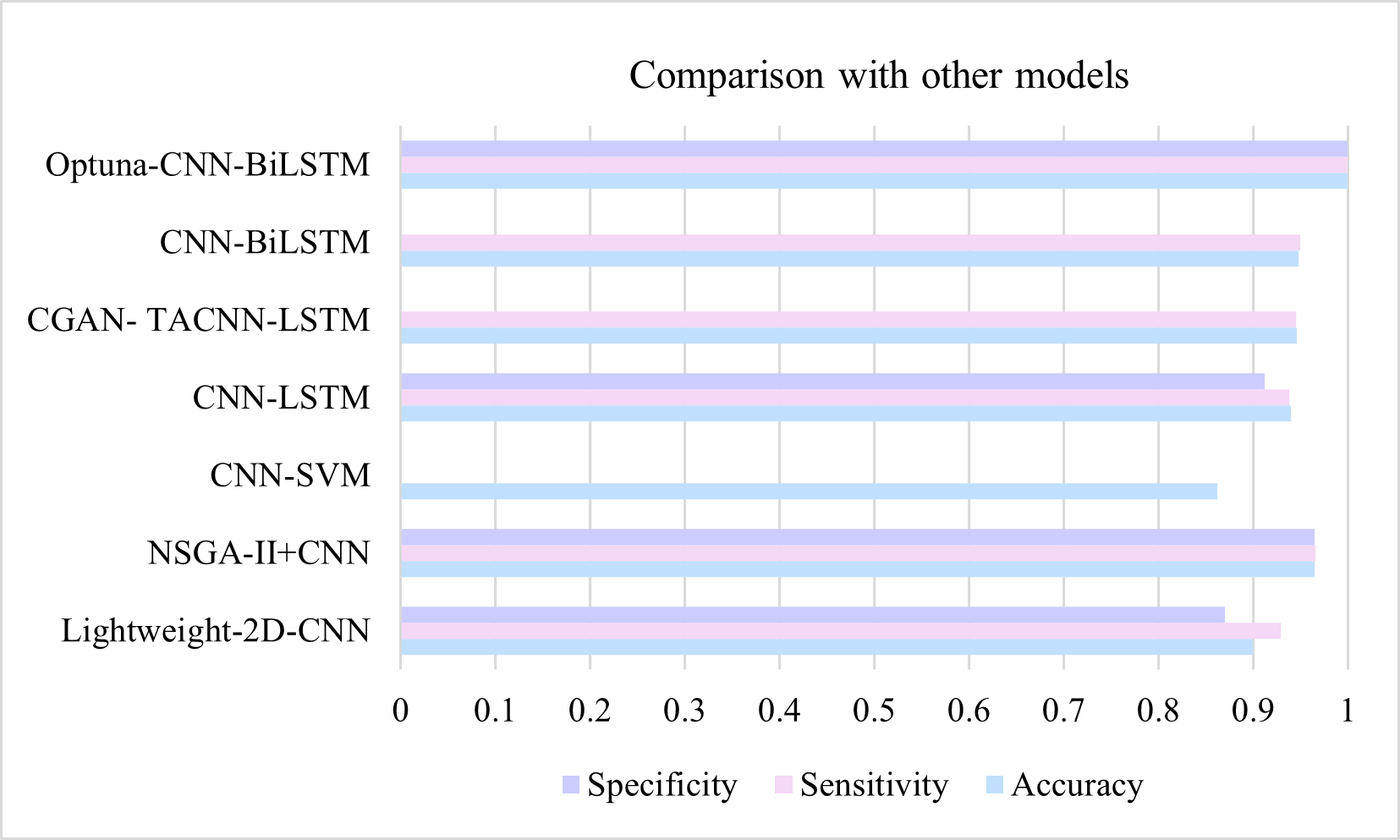

Supplement: Supplementary Figure 12 — Experimental results of different models. [file Image_12.tif]
